# Effect of frequency of clinic visits and medication pick‐up on antiretroviral treatment outcomes: a systematic literature review and meta‐analysis

**DOI:** 10.7448/IAS.20.5.21647

**Published:** 2017-07-21

**Authors:** Tsitsi Mutasa‐Apollo, Nathan Ford, Matthew Wiens, Maria Eugenia Socias, Eyerusalem Negussie, Ping Wu, Evan Popoff, Jay Park, Edward J. Mills, Steve Kanters

**Affiliations:** ^1^ College of Health Sciences, University of Zimbabwe, Harare, Zimbabwe; ^2^ AIDS and TB Unit, Ministry of Health and Child Care, Harare, Zimbabwe; ^3^ Department of HIV/AIDS, World Health Organization, Geneva, Switzerland; ^4^ Centre for Infectious Disease Epidemiology and Research, University of Cape Town, Cape Town, South Africa; ^5^ Precision Global Health, Department of Health Analytics, Vancouver, Canada; ^6^ School of Population and Public Health, University of British Columbia, Vancouver, Canada; ^7^ Interdisciplinary Graduate Program, University of British Columbia, Vancouver, Canada

**Keywords:** HIV Services; clinical visit frequency; antiretroviral therapy HIV/AIDS; systematic literature review; meta‐analysis

## Abstract

**Introduction**: Expanding and sustaining antiretroviral therapy (ART) coverage may require simplified HIV service delivery strategies that concomitantly reduce the burden of care on the health system and patients while ensuring optimal outcomes. We conducted a systematic review to assess the impact of reduced frequency of clinic visits and drug dispensing on patient outcomes.

**Methods**: As part of the development process of the World Health Organization antiretroviral (ARV) guidelines, we systematically searched medical literature databases for publications up to 30 August 2016. Information was extracted on trial characteristics, patient characteristics and the following outcomes: mortality, morbidity, treatment adherence, retention, patient and provider acceptability, cost and patients exiting the programme. When feasible, conventional pairwise meta‐analyses were conducted.

**Results and discussion**: Of 6443 identified citations, 21 papers, pertaining to 16 studies, were included in this review, with 11 studies contributing to analyses. Although analyses were feasible, they were limited by the sparse evidence base, despite the importance of the research area, and relatively low quality. Comparative analyses of eight studies reporting on frequency of clinic visits showed that less frequent clinic visits led to higher odds of being retained in care (odds ratio [OR]: 1.90; 95% CI: 1.21–2.99). No differences were found with respect to viral failure, morbidity or mortality; however, most estimates were favourable to reduced clinic visits. Reduced frequency of ARVs pick‐ups showed a trend towards better retention (OR: 1.93; 95% CI: 0.62–6.04). Strategies using community support tended to have better outcomes; however, their implementation varied, particularly by location. External validity may be questionable.

**Conclusions**: Our systematic review suggests that reduction of clinical visits (and likely ARVs pick‐ups) may improve clinical outcomes, and that they are a viable option to relieve health systems and reduce burden of care for PLHIV. Strategies aimed at reducing clinic visits or drug refill services should focus on stable patients who are virally suppressed, tolerant to their drug regimen and fully adherent. These strategies may be critical to the current changes taking place in HIV treatment policy; thus, due to the data limitations, further high quality research is needed to inform policy and programmatic interventions.

## Introduction

The global scale‐up of antiretroviral therapy (ART) has demonstrated major success by meeting the goal of 15 million people receiving life‐saving ART in 2015 [1]. However, the full potential of increased access to ART is undermined by high rates of treatment interruptions and loss to follow‐up following ART initiation [2–4]. In fact, recent estimates suggest that around half of patients are lost to care within four years of starting ART [5].

With trial evidence supporting initiation of ART as soon as possible following a positive HIV diagnosis, the pace of ART enrolment is likely to increase in the coming years [6,7]. ART is a life‐long treatment that requires repeated laboratory monitoring and clinic visits, which can result in substantial costs for patients and healthcare systems. At the same time, people living with HIV (PLHIV) often face multiple competing needs and logistical challenges that impede their ability to adhere to frequent (often monthly) clinic visits, particularly for individuals who have to travel long distances and incur direct and indirect costs [8,9]. Meeting these two challenges, enrolling more people on ART while improving retention in care for people on ART, will require adaptations to existing service delivery models. Reducing the frequency of both clinic visits and ARV refills has been proposed as one way to increase service efficiency and simplify care delivery for the majority of people on ART who are stable on treatment and require limited clinical support [10].

In order to support the development of World Health Organization (WHO) guidelines for simplified ART care delivery, we systematically reviewed the evidence to assess the impact of reduced frequency of clinic visits and drug dispensing on patient outcomes.

## Methods

### Eligibility criteria

Following a pre‐defined study protocol (Supplementary Appendix), we reviewed randomized trials, cohort studies, case control studies and cross‐sectional studies among PLHIV comparing less frequent clinic visits and drug refills against standard of care (SOC), as defined by the study; to be as inclusive as possible, no frequency was set a priori for either the intervention or comparator. Inclusion criteria in the form of a PICOS statement are provided in Table 1. Complex interventions that described a broader service adaptation such as decentralization of care from clinics to community sites were eligible for inclusion provided the adaptation included reduced frequency of visits and/or drug refills. All studies published prior to August 30, 2016, the date on which the search was completed, were considered eligible.

**Table 1:** Scope of the literature review in PICOS form

Criteria	Definition
Population	People living with HIV
Interventions	Less frequent clinic visits (intervals that are greater than the standard of care) Less frequent antiretroviral, cotrimoxazole and/or isoniazid preventative therapy pick‐ups (intervals that are greater than one month between pickups)
Comparator	Monthly clinic visits (or multiple visits per month) Monthly antiretroviral, cotrimoxazole and/or isoniazid preventative therapy pick‐ups (or multiple pick‐ups a month)
Outcomes	Mortality Morbidity Treatment adherence Retention (pre‐ and post‐ART initiation) Patient and provider acceptability Cost (including opportunity costs) Transfer out of programme
Study design	Randomized controlled trials and observational studies

### Search strategy

Using a broad search strategy (Supplementary Appendix), two reviewers (SK, MES) independently searched the following databases: EMBASE, MEDLINE and Cochrane Central Register of Controlled Trials. Conference abstracts provided through the EMBASE search, as well as the International AIDS conference, the annual Conference on Retroviruses and Opportunistic Infections, and the conference on HIV Pathogenesis, Treatment and Prevention (IAS) were also reviewed to determine if there were relevant studies that were recently completed. Additionally, hand searches of the bibliographies of published systematic reviews were performed.

### Study selection and data extraction

The same two reviewers (PW and SK) independently scanned all abstracts and potentially eligible articles. Where discrepancies in judgements between reviewers occurred, a third reviewer provided arbitration (TMA). Using a standard data extraction sheet, information was extracted on study characteristics, patient characteristics and the following outcomes: mortality, morbidity (WHO Stage III–IV defining illnesses or opportunistic infections), treatment adherence, retention, viral failure (defined as having detectable viral load after being virally suppressed according to a study defined threshold), patient and provider acceptability, cost and transfers out of programme. Among the extracted study characteristics were study inclusion criteria, which were used to determine the functional definition of stable HIV, a key concept which varied from study to study (see Table 2). The three components to stable HIV were being above a CD4 threshold, being virally suppressed and being adherent to ART. Many studies, particularly in low‐income settings, only required two of these components, but the underlying concept was that these were low‐risk, experienced patients. Another key definition was the frequency. More and less frequent were study defined. It was not the actual frequency that mattered, but the relative reduction of frequency that was of interest here. As can be seen in Table 2, there was some overlap in some of the shorter and longer frequencies. For example, the Selke et al. [31] study went from one to three months, while many of the others went from three to six months. Hence, the three‐month frequency represented more frequent for some studies, but less frequent for Selke et al.

**Table 2:** Characteristics of studies included in the principal systematic literature review

Study ID	Location	Study design	Inclusion criteria	Sample size	CD4 at baseline (cells/mm^3^)	Frequency	Approach	Outcomes	Conclusions
Babigumira et al. [19]	Kampala, Uganda	Retrospective cohort	Treatment experienced HIV patients with CD4 > 200 cells/µL and adherence >95% and no age restriction	829	Mean:268 (SD: 154)	Monthly clinic visits (SOC) vs. clinic visits every six months (PRP)	PRP: task‐shifting from primary care provider to pharmacists	Adherence Morbidity Patient acceptability Costs	The PRP is more cost‐effective program than the standard of care
Blair et al. [13]	USA	RCT	Treatment experienced HIV patients with CD4 > 200 cells/µL and adherent with no age restriction	110	NR	Clinic visits every three months vs. every six months	Reduced visit frequency within centralized HIV care	Mortality Morbidity Viral failure	Trend towards less break­through viremia and an increase in CD4 counts in patients seen more frequently in clinic
Buscher et al. [14]	USA	Retrospective cohort	HIV patients with viral load < 400 copies/mL and no age restriction	2171	Median:497 (IQR: 345–692)	Clinic visits every three or four months vs. every six months	Reduced visit frequency within centralized HIV care	Retention Viral failure	Clinicians are able to make safe decisions extending follow‐up intervals in persons with viral suppression
Grimsrud et al. [23]	Western Cape, South Africa	Programme data	Stable HIV patients on ART and ≥eighteen years of age	1860	NR	Drug refill every two months (SOC) vs. every four months	Reduced drug refill within the community adherence club programme	Retention Viral failure	These findings suggest that less frequent visits for stable ART patients should be evaluated as regular practice to alleviate unnecessary burden on patients and clinic resources
*Grimsrud et al. [22]	Western Cape, South Africa	Programme data	Stable HIV patients on ART and ≥eighteen years of age	8150	Median:130 (IQR: 64–197)	Clinic visits every two months vs. every twelve months	Community based adherence clubs (CACs)	LTFU Viral rebound	Stable primary‐care patients were successfully managed by CACs. Higher rates of retention and viral suppression were maintained in both men and women
Jaffar et al. [25]	Jinja, Uganda	Cluster‐randomized equivalence trial	Patients with WHO stage IV or late stage III disease or CD4‐cell counts fewer than 200 cells/µL on ART and ≥eighteen years of age	1453	Median:110 (IQR: 40–175)	Home‐based care vs. facility‐based care	Home‐based ART delivery by community health worker	Mortality Adherence Retention Viral failure	Home‐based HIV‐care strategy is as effective as clinic‐based strategy
Kipp et al. [26,27]	Karabole, Uganda	Prospective cohort	Treatment‐naïve patients with CD4 > 200 cells/µL and ≥eighteen years of age	385	Hospital: 136.1 (range: 3–477) Community: 146.4 (range: 1–578)	Monthly in facility‐based care (SOC) vs. every six months with community‐based care	Community‐based ART delivery (CBART)	Mortality Viral rebound	Acceptable rates of virologic suppression were achieved using existing rural clinic and community resources
Luque‐Fernandez et al. [28]	Cape Town, South Africa	Comparative Cohort	Treatment experienced HIV patients with CD4 > 200 cells/µL and ≥eighteen years of age	2829	Median:202 (IQR: 97–386)	Monthly clinic visits vs. every six months	CACs	Mortality Retention Viral rebound Costs	Patient adherence groups were found to be an effective model for improving retention and documented virologic suppression for stable patients in long term ART care
McGuire et al. [17,20]	Rural Malawi	Comparative Cohort	Treatment experienced HIV patients with CD4 > 300 cells/µL and >95% Adherence and ≥fifteen years of age	3818	Median:534 (IQR: 420–692)	Clinic visits every 1–two months vs. every six months drug pick‐up every three months	Clinical six month appointments and every three months drug refill (called the SMA programme)	Mortality Retention	Nearly 97% of patients remained in HIV care after twelve months of SMA program inclusion and those in care achieved satisfactory treatment outcomes
Muñoz‐Moreno et al. [29]	Spain	Comparative Cohort	Treatment naïve or experienced HIV patients and no age restriction	180	NR	Drug refill every three months vs. every six months	Reduced drug refill	Adherence	Less frequency in collecting medication does not have a negative impact on adherence and permits to maintain high levels of compliance
Selke et al. [31,32]	Western Kenya	RCT	Treatment experienced HIV patients living in the Kosirai with high adherence and ≥eighteen years of age	208	Intervention: 305 (IQR: 227–430) SOC: 278 (IQR:186–397)	SOC – monthly clinic visits Intervention – clinic visits every three months	Community care coordinator (CCC): Patients trained by HIV‐infected peers in three month intervals	Mortality Adherence Retention Morbidity Viral failure Patient provider acceptability	Community‐based care resulted in similar clinical outcomes as usual care but with half the number of clinic visits

RCT: randomized controlled trial; SOC: standard of care; PRP: pharmacy refill programme; PRP: Pharmacy‐only refill program; CBART: community‐based ART; FBART: facility‐based ART; IQR: Interquartile range. *This study was not included in the analyses due to non‐compatible data but did provide qualitative data to the review.

The validity of individual trials was assessed using the risk of bias instrument, endorsed by the Cochrane Collaboration [11], and risk of bias for observational studies was assessed using the National Institute for Health and Clinical Excellence checklist for observational studies [12]. The overall quality of the evidence was assessed using the GRADE framework.

### Statistical analyses

In situations with very limited data, a qualitative review was used as an alternative to quantitative analysis. When sufficient data were available for quantitative evidence synthesis, a conventional pairwise meta‐analysis was employed using the DerSimonian–Laird random‐effects model. The *I* ^2^ measure was used to gauge the degree of heterogeneity. When adjusted values were provided, steps were taken into account for these data. All results were presented with accompanying 95% confidence intervals (CIs). All analyses were performed using R version 3.1.2 (http://www.r-project.org/).

## Results and discussion

**Figure 1:**
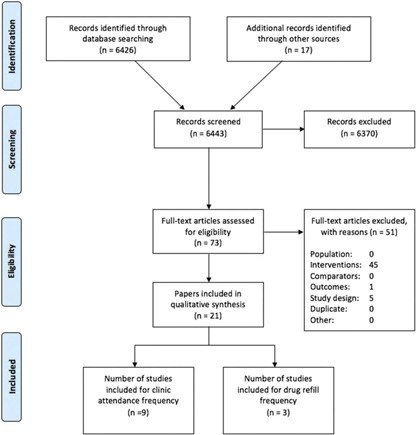
Flow chart of study screening. *Note*: The McGuire et al. study is included in both the clinic and drug refill frequency analyses. Therefore, 11 studies were included in the quantitative analyses.

Of 6443 citations identified through the systematic searches, 73 papers were selected for full‐text review and 21 papers, pertaining to 16 studies, met the inclusion criteria or provided sufficient information to be included in the qualitative review. The study selection process is summarized in Figure 1 and the list of studies included in the analyses is presented in Table 2.

Of the 16 included studies, 5 specifically investigated the role of reduced clinic visit and/or drug refill frequency [13–18], while 11 involved a change in the frequency of clinic attendance along with health worker task‐shifting [19–31]. Table 2 presents study characteristics for the 11 studies that were included in the quantitative analysis. The five other studies were only available for qualitative purposes. Of the analysed studies, only two were not restricted to stable HIV patients and one of these was the Jaffar et al. [25] RCT.

### Reduced clinical visit frequency: qualitative review

Three studies assessed reducing clinic visit frequency as the only programmatic change in a non‐comparative fashion. Admittedly, the first study, conducted in the United States in the mid‐1990s, is older and doesn't apply to today's HIV care climate. For this reason, it was not used in the meta‐analysis component. It assessed different clinic frequencies (eight, sixteen or twenty‐four weeks) within four randomized trials and concluded, based on missed drug toxicities, that reduced clinical care should not be recommended [16]. Importantly, these trials recruited patients with a mean baseline CD4 cell count of 46 cells/µL and took place when ART options were associated with greater frequency and severity of toxicity. More recently, a study conducted in Europe found that in clinically stable HIV patients (CD4 > 200 cells/mm^3^ and virally suppressed with no opportunistic infections), the risk of treatment failure was 1.6% (95% CI: 1.0–2.2) in the following six months and 3.6% (95% CI: 2.7–4.5) after twelve months [18]. Finally, a study conducted in London, England, found that reduced clinical visits through home medicine delivery led to comparable changes in CD4, viral suppression and adherence [15]. The latter two studies suggested that reduced clinical visit frequency might be a viable option for clinically stable HIV patients on a well‐tolerated ART regimen.

Two randomized trials, a prospective cohort and a retrospective cohort reported on a number of decentralized care models that brought care to communities or homes by utilizing community health workers or peers to deliver care, which effectively minimized the number of required clinic visits [25–27,31,32]. Importantly, these studies were not restricted to healthy, stable HIV patients but tended to be used by patients initiating ARV treatment.

Studies from the Democratic Republic of Congo, Malawi, Mozambique, South Africa and Haiti reported outcomes from community‐based care for stable HIV patients that included less frequent clinic visits [20–24,28,30,33]. In these models, patients are members of community groups who provide mutual adherence and social support and attend clinic services every three to six months to collect ART and receive a medical check‐up. There are various models within this class. One is community‐based adherence clubs (CACs), which are groups of up to 30 patients meeting every two months for less than an hour in order to pick‐up their medication and have a basic health assessment by the club facilitator (a peer educator and/or lay counsellor) [20,23,24,28]. Another model is community ART groups (CAGs), which was first piloted in the Tete province of Mozambique [21,33]. These are much smaller groups (4–6 patients), with each patient taking turns to go to the clinic to obtain the ART refills for the entire group and to have a clinic visit with a healthcare professional. In doing so, the frequency of clinic visits is reduced from once per month to once every four to six months. There are no comparative studies between CAG patients and patients using SOC within the same health system. Although comparisons of results within CAGs are favourable compared to those of the general HIV care programme in Mozambique, these studies suffer from important selection biases [21,33]. The CAG model has now been expanded to other regions, with favourable results reported in Haiti [30].

### Clinical visit frequency: quantitative synthesis

In total, eight studies informed the comparative analysis for frequency of clinic visits: three from Uganda, two from the USA and one from each Malawi, South Africa and Kenya [13,14,17,19,25,26,28,31]. These studies included 11,804 patients. Visit frequencies were classified as either frequent (usually every three months, sometimes monthly) or not frequent (generally every six months). One study compared the proportion of patients retained in care within the program participants to the proportion among eligible non‐participants. However, there was no information on sample size for the eligible non‐participators, and this was conservatively estimated to be one‐tenth of the participants of the intervention [17,20].

**Figure 2:**
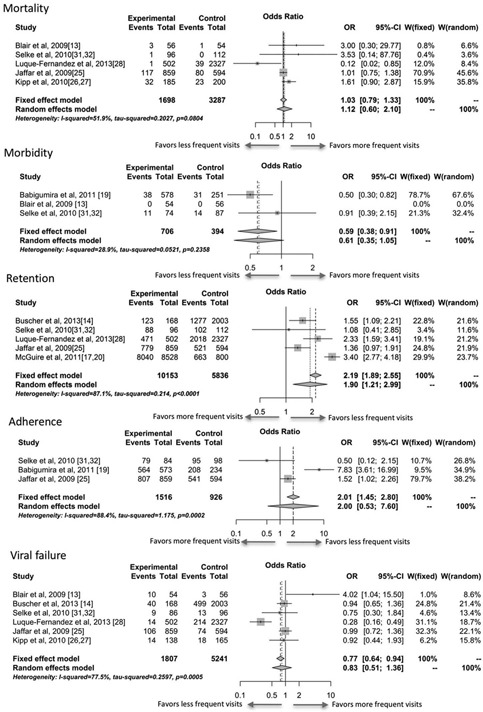
Meta‐analysis results comparing less frequent to more frequent clinic visits. Events for mortality were all cause; morbidity events were defined as developing a WHO Stage III–IV defining illness or opportunistic infection; retention events were persons completing a study period without discontinuation; adherence events were patients meeting the study defined adherence (>95% of pills taken, say); viral failure events was defined as a patient having detectable viral load after being suppressed.

Results are summarized in Figure 2. Mortality was comparable between groups with an odds ratio (OR) of 1.12 (95% CI: 0.60–2.10). Most of the events occurred in two randomized trials [17,25,26], which enrolled patients at treatment initiation rather than patients proven to be clinically stable. Consequently, patients tended to have a lower baseline CD4 at enrolment (median CD4 < 150 cells/mm^3^ across all arms), but no mortality differences in outcomes were reported between the different strategies in these trials. One study, from South Africa, reported a statistically significant reduction in mortality (1 death among the 502 patients in community clubs vs. 39 among 2327 patients in clinic care) [28]. No other study had an estimate suggesting a protective effect of reduced frequency of clinical visits.

Morbidity was poorly reported, with three studies reporting changes in morbidity and only two contributing to the analysis because the third study included no events. Although the meta‐analysis results tend towards a protective effect of less frequent clinic visits (OR: 0.61; 95% CI: 0.35–1.05), these results are driven by a single study [19]. In this study, the proportion of patients with an opportunistic infection (the marker of morbidity) was 6.6% in the pharmacy only refill program and 12.4% in the SOC program. However, the proportion of participants with opportunistic infections at baseline was imbalanced between the two arms (5.9% vs. 13.9%), which raises concern on the potential for selection bias.

Using a random‐effects approach, less frequent clinic visits led to higher odds of being retained in care (OR: 1.90; 95% CI: 1.21–2.99). All five studies had an estimate in favour of reduced clinic visit frequency, with the home‐based studies having the smallest estimated effect sizes. Although the fixed‐effects model for adherence suggested a significant improvement amongst less frequent visit groups, the analysis suffered from high heterogeneity, and therefore random‐effects model was selected. Note that results of analyses among CAC patients could not be synthesized with these data because they were presented as hazards ratios, but these did provide strong evidence that the risk of loss to follow‐up was lower in patients with reduced clinical visits (hazard ratio: 0.38; 95% CI: 0.32–0.45). In this model, lower clinic visit frequency was associated with a non‐statistically significant increase in adherence (OR: 2.00; 95% CI: 0.53–7.60).

The analysis of viral failure was also non‐significant with high heterogeneity. One randomized trial, which reduced frequency of clinic visits with no other concurrent intervention (in a developed country setting), showed a strong increase in the odds of viral failure in those with reduced frequency of clinic visits [13], while another study, using adherence clubs to reduce frequency of clinic visits in a South African setting, showed a reduced odds of viral failure [28].

### Drug refill frequency

Three studies were included in the evidence base on drug refill frequency [17,23,29]. One study evaluated adherence, one evaluated viral failure/mortality and two evaluated retention. A small study from Spain comparing one month to three‐month drug refill found that both groups achieved high levels of adherence (97%) [29]. Another study, from South Africa, reported on the effect of drug refill frequency (two vs. four months) on viral failure and mortality within select CACs [23]; this study found no statistical difference between groups with respect to either viral failure (OR: 1.03; 95% CI: 0.60–1.78) or mortality (no events), although the duration of observation only spanned four months.

**Figure 3:**
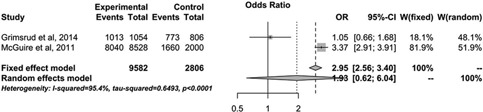
Meta‐analysis comparing retention among patients with less frequent drug refills and more frequent drug refills. ***Note***: Odds ratios of more than one favour less frequent visits (experimental) and odds ratios below one favour more frequent visits (control).

In the current meta‐analysis, there was a trend towards better retention among patients who received less frequent drug refills (OR: 1.93; 95% CI: 0.62–6.04) (Figure 3). One study, from Malawi [17], showed a strong effect, but the intervention comprised both reduced drug refill and clinic visits, making it difficult to independently assess each intervention. Nevertheless, in combination, these interventions resulted in a profound increase in overall retention, among those eligible for the intervention. In contrast, a study from South Africa showed no significant retention benefit among those with less frequent drug refills, although the period of observation was likely too short for any meaningful comparison between the two and four‐month refill groups [23].

### Quality of the evidence

The quality of evidence for frequency of clinic visits was rated as very low to low due to bias, principally due to a lack of adjustments for selection bias, and inconsistency. The complete GRADE tables are provided in the Supplementary Appendix. The different approaches to reducing frequency (with and without restrictions on clinical stability; with and without the addition of non‐clinical services) were considered, but no meta‐regression was possible given the sparseness of the data.

Although in the early stages of the ART scale‐up, many patients presented with advanced disease progression, the proportion of late diagnosis and late presenters has progressively improved over time [5]. Around half of the 15 million people who have initiated ART did so over four years ago [1,18]. With an increasing number of PLHIV on ART and progressively earlier treatment initiation thresholds, alternative strategies to provide quality care are needed. Frequent clinic visits are important for patients who present with advanced disease and become clinically unstable on treatment but appear to be of less benefit to clinically stable HIV patients [18].

The relatively high degree of clinical heterogeneity between studies highlights the need to consider the particular health system context in which these studies were implemented. In South Africa, community adherence groups were initiated in response to growing congestion and decreasing clinic retention. Patients considered to be clinically stable were given the opportunity to join community adherence groups, which not only decreases clinic congestion but also decreased mortality and viral failure rates [23,24,28]. On the other hand, in a study in the USA, where stable, adherent PLHIV either visited a clinic every three months or every six months, over a period of two years, there was a trend towards worse virological outcomes in the three‐month group [14]. Further, observed differences among studies included in this review might be explained by different strategies used to reduce clinic visit frequency (e.g. clinically stable HIV patients, clinically stable HIV patients with increased community support and increased community support regardless of clinical stability). As expected, results were consistently better among studies both requiring clinical stability and providing increased support, which allows for continued monitoring of patients outside the clinic environment. Differences in health systems only partially explain the high heterogeneity observed in the meta‐analyses. As was presented, there were notable differences in baseline characteristics that could not be adjusted for via meta‐regression using covariates due to the small number of studies found in this review.

This review is subject to several limitations. First, the majority of studies were observational and are subject to varying degrees of bias, the most important being selection bias in the form of selective inclusion of patients for the delayed clinic visits or drug refills. Since the comparator groups in some cases consisted of subjects less likely to be adherent or stable, reliable inferences become difficult and this limitation was reflected within the GRADE assessment. Second, the study may suffer from publication bias given that the evidence base was relatively small and that negative studies may be less likely to be published. Nonetheless, to this end, it is somewhat comforting that so few of the studies were retrospective in nature. Third, with respect to external validity, it is unlikely that these results extend beyond patients that are clinically stable. Fourth, the location of included studies varied, and the application of any strategy to reduce clinic visits or drug refills must be considered within a local context. Fifth, a large amount of heterogeneity was due to distinct general strategies employed in these studies: reduction of clinic or drug refill frequency without compensation based on clinical stability, reduction of clinic or drug refill frequency through increased community support based on clinical stability and reduction of clinic or drug refill frequency through increased community support without the requirement that patients are clinically stable. Finally, in some cases, the retention outcome was directly related to the intervention because less frequent visits lead to more occasions by which to identify that a person is lost to follow‐up.

Strengths of this review include a rigorous literature review process, and pre‐specified analytic techniques using random‐effects models that helped account for some of the heterogeneity observed between studies. Although many studies included retrospective cohort studies and programme evaluations and were subject to important biases not seen in randomized trials, they nevertheless were relatively free from the artificial interventional nature often characteristic of randomized trials. These studies, therefore, can be viewed as closer to programmatic experience.

### Assessment of the field

The findings of this review suggest that a reduced burden of care by reducing clinic visits may lead to improved outcomes, notably improved retention in care, and possibly also adherence, with no observed evidence of harmful effects. Reducing the frequency of drug refills also appeared to improve outcomes, although the evidence base was more limited. The limited evidence base for both these strategies certainly restrains the strength of the conclusions that can be drawn from these findings, but the positive trends indicate that well‐designed studies to properly assess these knowledge gaps would be a worthwhile endeavour. Further studies could also be tailored to answer more specific questions. The CAG model has shown promising results on a new continent (from Africa to America), but further studies could be used to determine the external validity of all reduction models. Research could also be used to better understand who to target and who benefits. A better understanding will also come from experience beyond the study level. We have discussed a variety of models, such as CAGs and CACs, that have shown benefits when conducted within a study, or with the assistance of organizations such as Médecin sans Frontiere, but it remains to be seen how such programs might work at a national level. These models are not without their potential pitfalls.

Reducing the frequency of clinic visits has potential benefit to the health systems that are overburdened in many high prevalence settings. Not only is it feasible, but it may also be more feasible than the status quo. The 2013 WHO Consolidated ART Guidelines recommended task shifting both within the healthcare system, and also to community peers [34]. Thus, community‐based interventions to support reduction in clinic visits may be seen as an extension of this prior recommendation. Reducing the frequency of drug refill, however, is logistically complex and requires a more medications to be dispensed during each visit, in turn requiring additional inventory. Given the reality of stock‐outs in the current environment of many resource limited countries, decisions on frequency of ARVs pick‐ups should be tailor‐made to match existing local capacity for medicine supply chain [35].

## Conclusions

In conclusion, this systematic literature review found that reduced frequency of clinic visits and drug refills may lead to improvements in program retention and patient outcomes among PLHIV. Strategies aimed at reducing clinic visits or drug refill services should focus on stable HIV patients who are virally suppressed, tolerant to their drug regimen and fully adherent. Further high‐quality research aimed specifically at providing evidence about the optimal frequency of clinic visits and ART refills, and the approaches most suitable for frequency modifications (community based vs. facility based) in different settings will be critical to inform policy and programmatic interventions.

## Competing interests

We declare that we have no conflicts of interest.

## Acknowledgements

The authors would like to thank the WHO guideline development group for their support and critical feedback: http://www.who.int/hiv/mediacentre/news/ARV-gdg2015/en/.

To access the supplementary material to this article please see http://doi.org/10.7448/IAS.20.5.21647 under Article Tools online.

## Supporting information

Supplementary material

Supplementary material

## References

[1] UNAIDS . “15 by 15”. A global target achieved. Geneva: UNAIDS; 2015.

[2] Kranzer K , Ford N . Unstructured treatment interruption of antiretroviral therapy in clinical practice: a systematic review. Trop Med Int Health. 2011;16(10): 1297–313. 21718394 10.1111/j.1365-3156.2011.02828.x

[3] Rosen S , Fox MP , Gill CJ . Patient retention in antiretroviral therapy programs in sub‐Saharan Africa: a systematic review. Plos Med. 2007;4(10): e298. 17941716 10.1371/journal.pmed.0040298 PMC2020494

[4] Boender TS , Sigaloff KC , McMahon JH , et al. Long‐term virological outcomes of first‐line antiretroviral therapy for HIV‐1 in low‐ and middle‐income countries: a systematic review and meta‐analysis. Clin Infect Dis. 2015;61 (9): 1453–61. 26157050 10.1093/cid/civ556 PMC4599392

[5] IeDEA‐WHO Collaboration: global analysis of retention in care in initial HIV care and treatment programmes in the IeDEA regions . 2015.

[6] Insight Start Study Group , Lundgren JD , Babiker AG , et al. Initiation of antiretroviral therapy in early asymptomatic HIV Infection. N Engl J Med. 2015;373(9): 795–807. 26192873 10.1056/NEJMoa1506816 PMC4569751

[7] Temprano Anrs Study Group , Danel C , Moh R , et al. A trial of early antiretrovirals and isoniazid preventive therapy in Africa. N Engl J Med. 2015;373(9): 808–22. 26193126 10.1056/NEJMoa1507198

[8] Govindasamy D , Ford N , Kranzer K . Risk factors, barriers and facilitators for linkage to antiretroviral therapy care: a systematic review. Aids. 2012;26 (16): 2059–67. 22781227 10.1097/QAD.0b013e3283578b9b

[9] Rachlis BS , Mills EJ , Cole DC . Livelihood security and adherence to antiretroviral therapy in low and middle income settings: a systematic review. Plos One. 2011;6(5): e18948. 21589911 10.1371/journal.pone.0018948 PMC3093377

[10] Duncombe C , Rosenblum S , Hellmann N , et al. Reframing HIV care: putting people at the centre of antiretroviral delivery. Trop Med Int Health. 2015;20(4): 430–47. 25583302 10.1111/tmi.12460 PMC4670701

[11] Higgins JP , Altman DG , Gotzsche PC , et al. The Cochrane Collaboration's tool for assessing risk of bias in randomised trials. BMJ. 2011; 343:d5928. doi: 10.1136/bmj.d5928. 10.1136/bmj.d5928 PMC3196245 22008217

[12] National Institute for Health and Care Excellence (NICE) . The gudelines manual: appendix C: methodology checklist: cohort studies. 2012 [cited 2015 Jun 12]. Available from: http://publications.nice.org.uk/the-guidelines-manual-appendices-bi-pmg6b/appendix-c-methodology-checklist-randomised-controlled-trials

[13] Blair D , Choudhary M , Morrison C . Effect of clinic visit frequency on HIV clinical care and virologic outcome. Philadelphia (PA): Infectious Diseases Society of America; 2009.

[14] Buscher A , Mugavero M , Westfall AO , Keruly J , Moore R , Drainoni ML , et al. The association of clinical follow‐up intervals in HIV‐infected persons with viral suppression on subsequent viral suppression. AIDS Patient Care STDS. 2013;27(8): 459–66. 23886048 10.1089/apc.2013.0105 PMC3739946

[15] Castelino S , Miah H , Auyeung V , Vogt F . Determination of the influence of home delivery of HIV therapy on virological outcomes and adherence. Int J STD AIDS. 2015;26(2): 93–7. 24733153 10.1177/0956462414530887

[16] Kendall MA , Andersen JW . van der Horst C. A reduced frequency visit schedule underreports adverse events that resulted in dose modifications or treatment discontinuations in HIV/AIDS clinical trials: ACTG DACS 207. Contemp Clin Trials. 2006;27(3): 287–94. 16545621 10.1016/j.cct.2006.02.001

[17] McGuire M , Pedrono G , Mukhuna B , Huckabee M , Heinzelmann A , Szumilin E , et al. Optimizing patient monitoring after the first year of ART: three years of implementing 6‐monthly clinical appointments in rural Malawi. Paper presented at: 6th IAS Conference on HIV Pathogenesis, Treatment and Prevention; 17‐20 July 2011.

[18] Reekie J , Mocroft A , Sambatakou H , Machala L , Chiesi A , van Lunzen J , et al. Does less frequent routine monitoring of patients on a stable, fully suppressed cART regimen lead to an increased risk of treatment failure? AIDS 2008;22(17): 2381–2390. 18981778 10.1097/QAD.0b013e328317a6eb

[19] Babigumira JB , Castelnuovo B , Stergachis A , Kiragga A , Shaefer P , Lamorde M , et al. Cost effectiveness of a pharmacy‐only refill program in a large urban HIV/AIDS clinic in Uganda. PLoS One. 2011;6(3): e18193. 21464895 10.1371/journal.pone.0018193 PMC3065481

[20] Bemelmans M , Baert S , Goemaere E , Wilkinson L , Vandendyck M , van Cutsem G , et al. Community‐supported models of care for people on HIV treatment in sub‐Saharan Africa. Trop Med Int Health. 2014;19(8): 968–77. 24889337 10.1111/tmi.12332

[21] Decroo T , Telfer B , Biot M , Maikere J , Dezembro S , Cumba LI , et al. Distribution of antiretroviral treatment through self‐forming groups of patients in Tete Province, Mozambique. J Acquir Immune Defic Syndr. 2011;56 (2):e39‐44. 10.1097/QAI.0b013e3182055138 21084990

[22] Grimsrud A , Lesosky M , Kalombo C , Bekker LG , Myer L . Implementation and operational research: community‐based adherence clubs for the management of stable antiretroviral therapy patients in Cape Town, South Africa: a cohort study. J Acquir Immune Defic Syndr. 2016;71(1): e16–23. 26473798 10.1097/QAI.0000000000000863

[23] Grimsrud A , Patten G , Sharp J , Myer L , Wilkinson L , Bekker LG . Extending dispensing intervals for stable patients on ART. J Acquir Immune Defic Syndr. 2014;66(2): e58–60. 24378724 10.1097/QAI.0000000000000098

[24] Grimsrud A , Sharp J , Kalombo C , Bekker LG , Myer L . Implementation of community‐based adherence clubs for stable antiretroviral therapy patients in Cape Town, South Africa. J Int AIDS Soc. 2015;18: 19984. 26022654 10.7448/IAS.18.1.19984 PMC4444752

[25] Jaffar S , Amuron B , Foster S , Birungi J , Levin J , Namara G et al. Rates of virological failure in patients treated in a home‐based versus a facility‐based HIV‐care model in Jinja, southeast Uganda: a cluster‐randomised equivalence trial. Lancet. 2009;374(9707): 2080–9. 19939445 10.1016/S0140-6736(09)61674-3 PMC2806484

[26] Kipp W , Konde‐Lule J , Saunders LD , Alibhai A , Houston S , Rubaale T et al. Antiretroviral treatment for HIV in rural Uganda: two‐year treatment outcomes of a prospective health centre/community‐based and hospital‐based cohort. PLoS One. 2012;7(7): e40902. 22815862 10.1371/journal.pone.0040902 PMC3398945

[27] Kipp W , Konde‐Lule J , Saunders LD , Alibhai A , Houston S , Rubaale T , et al. Results of a community‐based antiretroviral treatment program for HIV‐1 infection in Western Uganda. Curr HIV Res. 2010;8(2): 179–85. 20163349 10.2174/157016210790442722

[28] Luque‐Fernandez MA , van Cutsem G , Goemaere E , Hilderbrand K , Schomaker M , Mantangana N , et al. Effectiveness of patient adherence groups as a model of care for stable patients on antiretroviral therapy in Khayelitsha, Cape Town, South Africa. PLoS One. 2013;8(2): e56088. 23418518 10.1371/journal.pone.0056088 PMC3571960

[29] Muñoz‐Moreno JA , Fumaz CR , Ferrer MJ , Tuldra A , Andreu A , Bonafont X , et al. Influence of the frequency in the medication collection on adherence to antiretroviral therapy of HIV+ patients XV. Paper presented at: International AIDS Conference; 18‐22 July 2015.

[30] Naslund JA , Dionne‐Odom J , Junior Destine C , Jogerst KM , Renold Senecharles R , Jean Louis M et al. Adapting and Implementing a Community Program to Improve Retention in Care among Patients with HIV in Southern Haiti: “Group of 6”. AIDS Res Treat 2014; 2014: 137545. 25548659 10.1155/2014/137545 PMC4274858

[31] Selke HM , Kimaiyo S , Sidle JE , Vedanthan R , Tierney WM , Shen C , et al. Task‐shifting of antiretroviral delivery from health care workers to persons living with HIV/AIDS: clinical outcomes of a community‐based program in Kenya. J Acquir Immune Defic Syndr. 2010;55(4): 483‐90. 20683336 10.1097/QAI.0b013e3181eb5edb

[32] Wools‐Kaloustian KK , Sidle JE , Selke HM , Vedanthan R , Kemboi EK , Boit LJ , et al. A model for extending antiretroviral care beyond the rural health centre. J Int AIDS Soc. 2009;12: 22. 19788755 10.1186/1758-2652-12-22 PMC2762459

[33] Decroo T , Koole O , Remartinez D , dos Santos N , Dezembro S , Jofrisse M , et al. Four‐year retention and risk factors for attrition among members of community ART groups in Tete, Mozambique. Trop Med Int Health. 2014;19(5): 514–21. 24898272 10.1111/tmi.12278

[34] World Health Organization . Consolidated guidelines on the use of antiretroviral drugs for treating and preventing HIV infection: recommendations for a public health approach. Geneva: WHO; 2013. 24716260

[35] Sharma DC . Budget cuts threaten AIDS and tuberculosis control in India. Lancet. 2015;386(9997): 942. 26369456 10.1016/S0140-6736(15)00114-2

