# Supplementary material for: Effect of frequency of clinic visits and medication pick‐up on antiretroviral treatment outcomes: a systematic literature review and meta‐analysis
Source: J Int AIDS Soc. 2017 Jul 21;20(Suppl 4):21647. doi: 10.7448/IAS.20.5.21647 (PMC6192466; doi:10.7448/IAS.20.5.21647)
Supplement: Supplementary file 1 — Supplementary material [file JIA2-20-21647-s001.docx]

**Web Appendix**

**Criteria for study inclusion**

**Table A1** describes the PICOS (population, interventions, comparator, outcomes, study design) criteria used to guide the selection of studies that are included in this systematic literature review. Note that both research questions were captured in a single PICOS.

Table A1: Scope of the literature review in PICOS form

| **Criteria** | **Definition** |
| --- | --- |
| **Population** | - People living with HIV |
| **Interventions** | - Less frequent clinic visits (intervals that are greater than the standard of care) - Less frequent antiretroviral, co-trimoxazole and/or isoniazid preventative therapy pick-ups (intervals that are greater than one month between pickups) |
| **Comparator** | - Monthly clinic visits (or multiple visits per month) - Monthly antiretroviral, co-trimoxazole and/or isoniazid preventative therapy pick-ups (or multiple pick-ups a month) |
| **Outcomes** | - Mortality - Morbidity - Treatment adherence - Retention (pre- and post-ART initiation) - Patient and provider acceptability - Cost (including opportunity costs) - Transfer out of programme |
| **Study design** | Randomized controlled trials and observational studies |

**Search strategy**

**Table A2**: Systematic literature search strategy

| **c** | **Term** | **Comments** |
| --- | --- | --- |
|  | exp HIV/ OR exp HIV Infection/ | HIV/AIDS terms |
|  | (HIV Infections OR HIV?1* OR HIV?2* OR HIV infect* OR human immuno?deficiency virus OR human immune?deficiency virus).ti,ab. |  |
|  | ((human immun*) AND (deficiency virus)).ti,ab. |  |
|  | (acquired immuno?deficiency syndrome OR AIDS OR acquired immunedeficiency syndrome OR acquired immune deficiency).ti,ab. |  |
|  | ((acquired immun*) AND (deficiency syndrome)).ti,ab. |  |
|  | or/1-5 | **Population Final** |
|  | exp Appointments and Schedules/ | Frequency of visit and pickup |
|  | ((frequen* OR schedule* OR month* OR expect*) ADJ7 (visit* OR appoint* OR attend* OR follow-up*)).ti,ab,kw. |  |
|  | ((frequen* OR schedule* OR month* OR expect*) ADJ7 (pick?up* OR pick-up* OR deliver* OR pharma*)).ti,ab,kw. |  |
|  | exp Antiretroviral Therapy, Highly Active/ | ART and treatment |
|  | exp Anti-HIV Agents/ |  |
|  | exp Trimethoprim-Sulfamethoxazole Combination/ |  |
|  | (Septrin* or Bactrim* or Bactrimel* or Biseptol* or Cotrim* or Resprim* or Septra* or Sulfatrim* or Trisul* or Polytrim*).ti,ab. |  |
|  | co?trimoxazole.ti,ab. |  |
|  | (sxt or tmp-smx or tmp-smz or tmp-sulfa).ti,ab. |  |
|  | exp isoniazid/ |  |
|  | (Hydra* or Isovit* or Laniazid* or Nydrazid*).ti,ab. |  |
|  | (isonicotinylhydrazine or inh).ti,ab. |  |
|  | (or/7-8) OR (9 AND (or/10-18)) | **Intervention and comparators final** |
|  | (Randomized Controlled Trial or Controlled Clinical Trial).pt. | Randomized controlled trial terms |
|  | (Clinical Trial or Clinical Trial, Phase II or Clinical Trial, Phase III or Clinical Trial, Phase IV).pt. |  |
|  | Multicenter Study.pt. |  |
|  | Randomized Controlled Trial/ or Randomized Controlled Trials as Topic/ or "Randomized Controlled Trial (topic)"/ |  |
|  | Controlled Clinical Trial/ or Controlled Clinical Trials as Topic/ or "Controlled Clinical Trial (topic)"/ |  |
|  | Clinical Trial/ or Phase 2 Clinical Trial/ or Phase 3 Clinical Trial/ or Phase 4 Clinical Trial/ |  |
|  | Clinical Trials as Topic/ or Clinical Trials, Phase II as Topic/ or Clinical Trials, Phase III as Topic/ or Clinical Trials, Phase IV as Topic/ |  |
|  | "Clinical Trial (topic)"/ or "Phase 2 Clinical Trial (topic)"/ or "Phase 3 Clinical Trial (topic)"/ or "Phase 4 Clinical Trial (topic)"/ |  |
|  | (Nonrandom* or non random* or non-random* or quasi-random* or quasirandom*).ti,ab,hw. | Observational study design terms |
|  | cohort studies/ or cohort analysis/ |  |
|  | longitudinal studies/ or longitudinal study/ |  |
|  | prospective studies/ or prospective study/ |  |
|  | follow-up studies/ or follow up/ or followup studies/ |  |
|  | retrospective studies/ or retrospective study/ |  |
|  | observational study/ |  |
|  | quasi experimental methods/ or quasi experimental study/ |  |
|  | (quasi adj (experiment or experiments or experimental)).ti,ab. |  |
|  | ((non experiment or nonexperiment or non experimental or nonexperimental) adj3 (study or studies or design or analysis or analyses)).ti,ab. |  |
|  | or/20-37 | **Study design final** |
|  | 6 and 19 and 38 | **Complete search** |
|  | 39 not (review or letter or meta-analysis or case report* or case series or posters or News or Newspaper article or meeting abstracts or lectures or interview or historical article or handbooks or guidelines or guidebooks or essays or editorial or comment or clinical conference or catalogs).pt. | Remove unwanted publication types |

**List of included studies**

**Table A3:** List of included studies and all included papers pertaining to those studies

| **Study ID** | **Author** | **Year** | **Title** | **Research Qestion** |
| --- | --- | --- | --- | --- |
| ACTG DACS 207 | Kendall, MA et al^1^ | 2006 | A reduced frequency visit schedule underreports adverse events that resulted in dose modifications or treatment discontinuations in HIV/AIDS clinical trials: ACTG DACS 207 | Clinic visit frequency |
| Babigumira et al, 2011 | Babigumira, JB et al^2^ | 2011 | Cost effectiveness of a pharmacy-only refill program in a large urban HIV/AIDS clinic in Uganda | Clinic visit and drug refill frequency |
| Blair et al, 2009 | Blair, D et al^3^ | 2009 | Effect of Clinic Visit Frequency on HIV Clinical Care and Virologic Outcome | Clinic visit frequency |
| Buscher et al, 2013 | Buscher, A et al^4^ | 2013 | The association of clinical follow-up intervals in HIV-infected persons with viral suppression on subsequent viral suppression | Clinic visit frequency |
| Catelino et al, 2015 | Catelino, S et al^5^ | 2015 | Determination of the influence of home delivery of HIV therapy on virological outcomes and adherence | Clinic visit frequency |
| Decroo et al, 2011 | Decroo, T et al^6^ | 2011 | Distribution of antiretroviral treatment through self-forming groups of patients in Tete province, Mozambique | Clinic visit frequency |
|  | Decroo, T et al^7^ | 2014 | Four-year retention and risk factors for attrition among members of community ART groups in Tete, Mozambique |  |
| Grimsrud et al, 2014 | Grimsrudm A et al^8^ | 2014 | Extending dispensing intervals for stable patients on ART | Drug refill frequency |
| Grimsrud et al, 2015 | Grimsrudm A et al^9^ | 2015 | Implementation of community-based adherence clubs for stable antiretroviral therapy patients in Cape Town, South Africa | Clinic visit frequency |
|  | Grimsrudm A et al^10^ | 2016 | Community-Based Adherence Clubs for the Management of Stable Antiretroviral Therapy Patients in Cape Town, South Africa: A Cohort Study | Clinic visit frequency |
| Group of 6 | Naslund, JA et al^11^ | 2014 | Adapting and Implementing a Community Program to Improve Retention in Care among Patients with HIV in Southern Haiti: "Group of 6" | Clinic visit frequency |
| Jinja Trial, 2009 | Jaffar, S et al^12^ | 2009 | Rates of virological failure in patients treated in a home-based versus a facility-based HIV-care model in Jinja, southeast Uganda: a cluster-randomised equivalence trial | Clinic visit frequency |
| Kipp et al, 2010 | Kipp, W et al^13^ | 2010 | Results of a community-based antiretroviral treatment program for HIV-1 infection in Western Uganda | Clinic visit frequency |
|  | Kipp, W et al^14^ | 2012 | Antiretroviral treatment for HIV in rural Uganda: two-year treatment outcomes of a prospective health centre/community-based and hospital-based cohort |  |
| Luque-Fernandez et al, 2013 | Luque-Fernandez, MA et al^15^ | 2013 | Effectiveness of patient adherence groups as a model of care for stable patients on antiretroviral therapy in Khayelitsha, Cape Town, South Africa | Clinic visit frequency |
| Many | Bemelmans, M et al^16^ | 2014 | Community-supported models of care for people on HIV treatment in sub-Saharan Africa | Clinic visit frequency |
| McGuire et al, 2011 | McGuire, M et al | 2011 | Optimizing patient monitoring after the first year of ART: three years of implementing 6-monthly clinical appointments in rural Malawi | Clinic visit frequency |
| Muñoz-Moreno et al, 2015 | Muñoz-Moreno J A^17^ | 2015 | Influence of the frequency in the medication collection on adherence to antiretroviral therapy of HIV+ patients | Drug refill frequency |
| Reekie et al, 2008 | Reekie, J et al^18^ | 2008 | Does less frequent routine monitoring of patients on a stable, fully suppressed cart regimen lead to an increased risk of treatment failure? | Clinic visit frequency |
| Selke et al, 2010 | Selke, HM et al^19, 20^ | 2010 | Task-shifting of antiretroviral delivery from health care workers to persons living with HIV/AIDS: Clinical outcomes of a community-based program in Kenya | Clinic visit frequency |
|  | Wools-Kaloustian, KK et al | 2009 | A model for extending antiretroviral care beyond the rural health centre |  |

**List of studies excluded** **after full-text review**

Note that many of the papers listed below have been excluded on interventions because the reviewers included an extensive amount of papers pertaining to the improvement of retention for full text reviews. These studies used other interventions that did not reduce the clinical or pharmacy refill frequency.

**Table A4: List of studies excluded at full text screening**

| **First Author** | | **Title** | | **Year** | **Reason** |
| --- | --- | --- | --- | --- | --- |
| Elbirt, D et al | | Direct monthly highly active antiretroviral therapy supply - a method to increase patient's adherence and outcome. Experience of one AIDS centre in Israel | 2014 | Interventions – does not investigate clinical frequency | |
| Braitstein, P et al | | A clinician-nurse model to reduce early mortality and increase clinic retention among high-risk HIV-infected patients initiating combination antiretroviral treatment | 2012 | Interventions – does not investigate clinical frequency | |
| Crawford, TN | | Poor retention in care one-year after viral suppression: A significant predictor of viral rebound | 2014 | Interventions – does not investigate clinical frequency | |
| Davies, O et al | | Validation of the rationalisation of routine blood tests and visits for HIV-1-infected individuals | 2014 | Interventions – does not investigate clinical frequency | |
| Fleishman, JA et al | | Associations between outpatient and inpatient service use among persons with HIV infection: A positive or negative relationship? | 2008 | Interventions – does not investigate clinical frequency | |
| Frei, CR et al | | Comparative value of four measures of retention in expert care in predicting clinical outcomes and health care utilization in HIV patients | 2013 | Interventions – does not investigate clinical frequency | |
| Gardner, LI et al | | Enhanced personal contact with HIV patients improves retention in primary care: A randomized trial in 6 us HIV clinics | 2014 | Interventions – does not investigate clinical frequency | |
| Jonas, A et al | | National art monitoring system enables robust transfer-adjusted retention analyses in Namibia | 2014 | Interventions – does not investigate clinical frequency | |
| Jones, D et al | | Enhancing HIV medication adherence in India | 2013 | Interventions – does not investigate clinical frequency | |
| Juma, JM et al | | Monitoring prevention or emergence of HIV drug resistance: Results of a population-based foundational survey of early warning indicators in mainland Tanzania | 2014 | Interventions – does not investigate clinical frequency | |
| Konkle-Parker, DJ et al | | Effects of an intervention addressing information, motivation, and behavioral skills on HIV care adherence in a southern clinic cohort | 2014 | Interventions – does not investigate clinical frequency | |
| Kunutsor, S et al | | Clinic attendance for medication refills and medication adherence amongst an antiretroviral treatment cohort in Uganda: A prospective study | 2010 | Interventions – does not investigate clinical frequency | |
| Kwena, ZA et al | | The feasibility and economic impact of time designated appointment system in a busy HIV care clinic | 2014 | Interventions – does not investigate clinical frequency | |
| Lee, L et al | | Improved retention in care for Ugandan youth living with HIV utilizing a youth-targeted clinic at entry to adult care: Outcomes and implications for a transition model | 2015 | Interventions – does not investigate clinical frequency | |
| Lemly, D et al | | Factors related to medical appointment attendance after childbirth among HIV-infected women in the Paris region | 2007 | Outcomes | |
| Macleod, WB et al | | The feasibility of using screening criteria to reduce clinic visits for stable patients on antiretroviral therapy in south Africa | 2013 | Interventions – does not investigate clinical frequency | |
| Maselle, E et al | | Reducing turnaround time for laboratory test results does not improve retention of stable HIV-infected adults on pov program: Experience from Uganda | 2014 | Interventions – does not investigate clinical frequency | |
| Mcnaghten, AD et al | | Differences in prescription of antiretroviral therapy in a large cohort of HIV-infected patients | 2003 | Interventions – does not investigate clinical frequency | |
| Mcnairy, ML et al | | Retention of HIV-infected children on antiretroviral treatment in HIV care and treatment programs in Kenya, Mozambique, Rwanda, and Tanzania | 2013 | Interventions – does not investigate clinical frequency | |
| Mugavero, MJ et al | | Measuring retention in HIV care: The elusive gold standard | 2012 | Interventions – does not investigate clinical frequency | |
| Mugusi, F et al | | Enhancing adherence to antiretroviral therapy at the HIV clinic in resource constrained countries; the Tanzanian experience | 2009 | Interventions – does not investigate clinical frequency | |
| Mwachari, CW et al | | Mortality and burden of disease in a cohort of HIV-seropositive adults in Nairobi, Kenya | 2004 | Study design | |
| Naar-King, S et al | | Ancillary services and retention of youth in HIV care | 2007 | Interventions – does not investigate clinical frequency | |
| Nachega, JB et al | | Antiretroviral therapy adherence, virologic and immunologic outcomes in adolescents compared with adults in southern Africa | 2009 | Interventions – does not investigate clinical frequency | |
| Nachega, JB et al | | Adherence to non-nucleoside reverse transcriptase inhibitor-based HIV therapy and virologic outcomes | 2007 | Interventions – does not investigate clinical frequency | |
| Nakiwogga-Muwanga, A et al | | Factors before enrolment are associated with being removed from a pharmacy-only refill programme at a large urban HIV/AIDS clinic, Uganda | 2014 | Study design | |
| Ndyanabo, A et al | | Measures of art adherence associated with virological failure in Rakai, Uganda | 2014 | Study design | |
| Ostrowski, SR et al | | Low-level viremia and pro-viral DNA impede immune reconstitution in HIV-1-infected patients receiving highly active antiretroviral therapy | 2005 | Interventions – does not investigate clinical frequency | |
| Romih, V et al | | Frequency of HIV-1 viral load monitoring of patients initially successfully treated with combination antiretroviral therapy | 2010 | Interventions – does not investigate clinical frequency | |
| Seguy, N et al | | Evaluation of the consistency of refills for antiretroviral medications in two hospitals in the state of Rio de Janeiro, Brazil | 2007 | Interventions – does not investigate clinical frequency | |
| Townsend, ML et al | | Association between pharmacy medication refill-based adherence rates and cd4 count and viral-load responses: A retrospective analysis in treatment-experienced adults with HIV | 2007 | Study design | |
| Tripathi, A et al | | The impact of retention in early HIV medical care on viro-immunological parameters and survival: A statewide study | 2011 | Interventions – does not investigate clinical frequency | |
| Wringe, A et al | | Doubts, denial and divine intervention: Understanding delayed attendance and poor retention rates at a HIV treatment programme in rural Tanzania | 2009 | Study design | |
| Yehia, BR et al | | Measures of retention in HIV care differ in their associations with clinical HIV outcomes | 2014 | Interventions – does not investigate clinical frequency | |
| Rebeiro, P et al | | HIV Testing and the Continuum of Care in the Industrialized World | 2015 | Interventions – does not investigate clinical frequency | |
| Adams, J et al | | HIV Care Continuum for Postpartum Women in Philadelphia: Barriers and Facilitators | 2015 | Interventions – does not investigate clinical frequency | |
| Colasanti, Jonathan | | A Longitudinal Approach to Retention and Virologic Suppression Across the HIV Care Continuum | 2015 | Interventions – does not investigate clinical frequency | |
| Yehia, B et al | | The HIV Treatment Cascade: Is There More To the Story? | 2014 | Interventions – does not investigate clinical frequency | |
| Belaunzaran-Zamudio, P. et al | | Cd4 and hiv-vl monitoring practices and costs in patients on art in latin america and the caribbean | 2015 | Interventions – does not investigate clinical frequency | |
| Giacomelli, A. et al | | Factors involved in continuance of atazanavir-based regimens: Results from a cohort of hiv1-positive patients | 2016 | Interventions – does not investigate clinical frequency | |
| Go, V. F. et al | | Efficacy of a multi-level intervention to reduce injecting and sexual risk behaviors among hiv-infected people who inject drugs in vietnam: A four-arm randomized controlled trial | 2015 | Interventions – does not investigate clinical frequency | |
| Ho, C. P. et al | | Factors associated with missed psychiatry visits in an urban hiv clinic | 2015 | Interventions – does not investigate clinical frequency | |
| Janssen, S. et al | | Factors associated with retention to care in an hiv clinic in gabon, central africa | 2015 | Interventions – does not investigate clinical frequency | |
| Kimeu, M. et al | | The relationship between adherence to clinic appointments and year-one mortality for newly enrolled hiv infected patients at a regional referral hospital in western kenya, january 2011-december 2012 | 2016 | Interventions – does not investigate clinical frequency | |
| Kwena, Z. A. et al | | The feasibility, time savings and economic impact of a designated time appointment system at a busy hiv care clinic in kenya: A randomized controlled trial | 2015 | Interventions – does not investigate clinical frequency | |
| Laurens, M. B. et al | | Tscq study: A randomized, controlled, open-label trial of daily trimethoprim-sulfamethoxazole or weekly chloroquine among adults on antiretroviral therapy in malawi: Study protocol for a randomized controlled trial | 2016 | Interventions – does not investigate clinical frequency | |
| Leblanc, J. et al | | The impact of nurse-driven targeted hiv screening in 8 emergency departments: Study protocol for the dici-vih cluster-randomized two-period crossover trial | 2016 | Interventions – does not investigate clinical frequency | |
| Lubega, M. et al | | Effect of community support agents on retention of people living with hiv in pre-antiretroviral care: A randomized controlled trial in eastern uganda | 2015 | Interventions – does not investigate clinical frequency | |
| Mugo, P. M. et al | | Effect of text message, phone call, and in-person appointment reminders on uptake of repeat hiv testing among outpatients screened for acute hiv infection in kenya: A randomized controlled trial | 2016 | Interventions – does not investigate clinical frequency | |
| Sabin, C. et al | | Investigating associations between a new measure of engagement in-care and clinical outcomes in the uk collaborative hiv cohort (uk chic) study | 2015 | Interventions – does not investigate clinical frequency | |
| Stankievich, E. et al | | Utility of mobile communication devices as a tool to improve adherence to antiretroviral treatment in hiv-infected children and adolescents in argentina | 2015 | Interventions – does not investigate clinical frequency | |
| Traore, I. T. et al | | Hiv prevention and care services for female sex workers: Efficacy of a targeted community-based intervention in burkina faso | 2015 | Interventions – does not investigate clinical frequency | |

**Table A5:** Patient characteristics across the 8 trials included in the principal analysis

| **Study ID** | **Intervention frequency** | **Level of care** | **Age**  **mean (SD)** | **Males**  **n (%)** | **AIDS-defining illness n (%)** | **Baseline CD4 (cells/µl)**  **Mean (SD)** |
| --- | --- | --- | --- | --- | --- | --- |
| Babigumira et al, 2011^2^ | Clinic visit every month | Outpatient clinic | 38.8 (7.5) | 100/251 (39.8%) | 35/251 (13.9%) | 218 (160) |
|  | Clinic visit every 6 months | Dispensary | 35.9 (7.5) | 253/578 (43.8%) | 34/578 (5.8%) | 292 (145) |
| Blair et al, 2009^3^ | Clinic visit every 3 months | Clinic | -- | -- | -- | -- |
|  | Clinic visit every 6 months | Clinic | -- | -- | -- | -- |
| Buscher et al, 2013^4^ | Clinic visit every 3 months | Primary health care clinic | 47 (10) | 973/1429 (68%) | -- | 497^§^ |
|  | Clinic visit every 4 months | Primary health care clinic | 47 (10) | 412/574 (72%) | -- | 497^§^ |
|  | Clinic visit every 6 months | Primary health care clinic | 47 (10) | 127/168 (76%) | -- | 497^§^ |
| Grimsrud et al, 2014^8^ | 4 month pick-up | Adherence clubs | -- | -- | -- | -- |
|  | 2 month pick-up | Adherence clubs | -- | -- | -- | -- |
| Grimsrud et al, 2016^10^ | Clinic visit every 2 months | Standard of care – health clinic | 33.2 (28.0 – 39.8) ^†^ | 1946 (31.2%) | -- | 128 (61 – 198)^†^ |
|  | Clinic visit every 12 months | Adherence clubs – community health | 33.9 (29.4 – 39.8) ^†^ | 624 (29.5%) | -- | 134 (73 – 195) ^†^ |
| Jinja Trial, 2009^12^ | Clinic visit every 6 months | CBART | 37 (32 – 44)^†^ | 234 (27%) | 71 (8%) | 99 (34 – 162)^†^ |
|  | Clinic visit every 3 months | FBART | 38 (33 – 44)^†^ | 188 (32%) | 47 (8%) | 122 (30 – 167)^†^ |
| Kipp et al, 2010^13, 14^ | Clinic visit every 6 months | CBART | 36.8 (8.9) | 76 (41%) | -- | 137 (81 – 193)^†^ |
|  | Clinic visit every month | FBART | 34.8 (11.5) | 87 (44%) | -- | 131 (66 – 200)^†^ |
| Luque-Fernandez et al, 2013^15^ | Clinic visit 3 every months | Community health centre | 37.5 (28.5 – 39)^†^ | 31 (28%) | 22 (20%) | 278 |
|  | Clinic visit every monthly | Community health centre | 38.7 (28.5 – 39)^†^ | 25 (26%) | 19 (20%) | 305 |
| McGuire et al, 2011^16, 21^ | Clinic visit every 6 months and refill 3 every months | HIV clinic | -- | 1184 (31%) | -- | 534^§^ |
|  | Clinic visit every 3 months and refill monthly | HIV clinic | -- | -- | -- | -- |
| Muñoz-Moreno et al, 2004^17^ | Refill every 3 months | Primary healthcare | -- | -- | -- | -- |
|  | Refill every 1 month | Primary healthcare | -- | -- | -- | -- |
| Selke et al, 2010^19, 20^ | Clinic visit every 3 months | HIV clinic | 37.5 (9.5) | 25 (26%) | 19 (20) | 305 (227 – 430)^†^ |
|  | Clinic visit every month | HIV clinic | 38.5 (9.9) | 31 28%) | 22 (20) | 278 (186 – 397)^†^ |

*†: Median and interquartile interval; §: median; CBART: community based ART; FBART: facility based ART; SD: standard deviation.*

Table A6: Cochrane risk of bias quality assessment for randomized controlled trials

| Trial | Sequence generation | Allocation concealment | Blinding | Incomplete outcome data | Selective outcome reporting | Other sources of bias |
| --- | --- | --- | --- | --- | --- | --- |
| Blair et al, 2009^3^ | Unclear | Unclear | High (not blinded) | Unclear | Low | Small sample/selection bias |
| Jinja Trial, 2009^12^ | Low | Low | High (not blinded) | Low | Low | Cluster design leads to poor balance of patient characteristics |
| Selke et al, 2010^19, 20^ | Low | Low | Moderate/high | Low | Low | Small sample/selection bias |

Table A7: Risk of bias assessment for observational studies using the NICE checklist

| Trial | Participants blind to treatment allocation | Provider blind to treatment allocation | Equal follow-up time | Comparable treatment completion | Comparable outcome data | Appropriate length of follow-up time | Precise outcome definitions | Valid and reliable outcome measures | Investigators blind to allocation | Investigators blind to other confounding factors |
| --- | --- | --- | --- | --- | --- | --- | --- | --- | --- | --- |
| Babigumira et al, 2011^2^ | No | No | No | Yes | Yes | Yes | Yes | Yes | No | No |
| Buscher et al, 2013^4^ | No | No | Yes | Yes | Yes | Yes | Yes | Yes | No | No |
| Grimsrud et al, 2014^8^ | No | No | Yes | Yes | Yes | Yes | Yes | Yes | No | No |
| Grimsrud et al, 2016^10^ | No | No | Yes | Yes | Yes | Yes | Yes | Yes | No | No |
| Kipp et al, 2010^13, 14^ | No | No | Yes | Yes | Yes | Yes | Yes | Yes | No | No |
| Luque-Fernandez et al, 2013^15^ | No | No | No | Yes | Yes | Yes | Yes | Yes | No | No |
| McGuire et al, 2011^16, 21^ | No | No | No | Yes | Yes | Yes | Yes | Yes | No | No |
| Muñoz-Moreno et al, 2004^17^ | No | No | Yes | Unclear | Unclear | Yes | Unclear | Unclear | No | No |

1. Kendall MA, Andersen JW, van der Horst C. A reduced frequency visit schedule underreports adverse events that resulted in dose modifications or treatment discontinuations in HIV/AIDS clinical trials: ACTG DACS 207. *Contemporary Clinical Trials* 2006; **27** (3): 287-94.

2. Babigumira JB, Castelnuovo B, Stergachis A et al. Cost effectiveness of a pharmacy-only refill program in a large urban HIV/AIDS clinic in Uganda. *PLoS One* 2011; **6** (3): e18193.

3. Blair D, Choudhary M, Morrison C. Effect of clinic visit frequency on HIV clinical care and virologic outcome. Infectious Diseases Society of America; Philadelphia, PA, USA2009.

4. Buscher A, Mugavero M, Westfall AO et al. The association of clinical follow-up intervals in HIV-infected persons with viral suppression on subsequent viral suppression. *AIDS Patient Care STDS* 2013; **27** (8): 459-66.

5. Castelino S, Miah H, Auyeung V, Vogt F. Determination of the influence of home delivery of HIV therapy on virological outcomes and adherence. *Int J STD AIDS* 2015; **26** (2): 93-7.

6. Decroo T, Telfer B, Biot M et al. Distribution of antiretroviral treatment through self-forming groups of patients in Tete Province, Mozambique. *J Acquir Immune Defic Syndr* 2011; **56** (2): e39-44.

7. Decroo T, Koole O, Remartinez D et al. Four-year retention and risk factors for attrition among members of community ART groups in Tete, Mozambique. *Trop Med Int Health* 2014; **19** (5): 514-21.

8. Grimsrud A, Patten G, Sharp J, Myer L, Wilkinson L, Bekker LG. Extending dispensing intervals for stable patients on ART. *J Acquir Immune Defic Syndr* 2014; **66** (2): e58-60.

9. Grimsrud A, Sharp J, Kalombo C, Bekker LG, Myer L. Implementation of community-based adherence clubs for stable antiretroviral therapy patients in Cape Town, South Africa. *J Int AIDS Soc* 2015; **18** 19984.

10. Grimsrud A, Lesosky M, Kalombo C, Bekker LG, Myer L. Implementation and Operational Research: Community-Based Adherence Clubs for the Management of Stable Antiretroviral Therapy Patients in Cape Town, South Africa: A Cohort Study. *J Acquir Immune Defic Syndr* 2016; **71** (1): e16-23.

11. Naslund JA, Dionne-Odom J, Junior Destine C et al. Adapting and Implementing a Community Program to Improve Retention in Care among Patients with HIV in Southern Haiti: "Group of 6". *AIDS Res Treat* 2014; **2014** 137545.

12. Jaffar S, Amuron B, Foster S et al. Rates of virological failure in patients treated in a home-based versus a facility-based HIV-care model in Jinja, southeast Uganda: a cluster-randomised equivalence trial. *Lancet* 2009; **374** (9707): 2080-9.

13. Kipp W, Konde-Lule J, Saunders LD et al. Results of a community-based antiretroviral treatment program for HIV-1 infection in Western Uganda. *Curr HIV Res* 2010; **8** (2): 179-85.

14. Kipp W, Konde-Lule J, Saunders LD et al. Antiretroviral treatment for HIV in rural Uganda: two-year treatment outcomes of a prospective health centre/community-based and hospital-based cohort. *PLoS One* 2012; **7** (7): e40902.

15. Luque-Fernandez MA, Van Cutsem G, Goemaere E et al. Effectiveness of patient adherence groups as a model of care for stable patients on antiretroviral therapy in Khayelitsha, Cape Town, South Africa. *PLoS One* 2013; **8** (2): e56088.

16. Bemelmans M, Baert S, Goemaere E et al. Community-supported models of care for people on HIV treatment in sub-Saharan Africa. *Trop Med Int Health* 2014; **19** (8): 968-77.

17. Muñoz-Moreno JA, Fumaz CR, Ferrer MJ et al. Influence of the frequency in the medication collection on adherence to antiretroviral therapy of HIV+ patients XV International AIDS Conference; Bangkok, Thailand2004.

18. Reekie J, Mocroft A, Sambatakou H et al. Does less frequent routine monitoring of patients on a stable, fully suppressed cART regimen lead to an increased risk of treatment failure? *Aids* 2008; **22** (17): 2381-90.

19. Selke HM, Kimaiyo S, Sidle JE et al. Task-shifting of antiretroviral delivery from health care workers to persons living with HIV/AIDS: clinical outcomes of a community-based program in Kenya. *J Acquir Immune Defic Syndr* 2010; **55** (4): 483-90.

20. Wools-Kaloustian KK, Sidle JE, Selke HM et al. A model for extending antiretroviral care beyond the rural health centre. *J Int AIDS Soc* 2009; **12** 22.

21. McGuire M, Pedrono G, Mukhuna B et al. Optimizing patient monitoring after the first year of ART: three years of implementing 6-monthly clinical appointments in rural Malawi. 6th IAS Conference on HIV Pathogenesis, Treatment and Prevention; Rome, Italy2011.
