# Supplementary material for: Effect of frequency of clinic visits and medication pick‐up on antiretroviral treatment outcomes: a systematic literature review and meta‐analysis
Source: J Int AIDS Soc. 2017 Jul 21;20(Suppl 4):21647. doi: 10.7448/IAS.20.5.21647 (PMC6192466; doi:10.7448/IAS.20.5.21647)
Supplement: Supplementary file 2 — Supplementary material [file JIA2-20-21647-s002.doc]

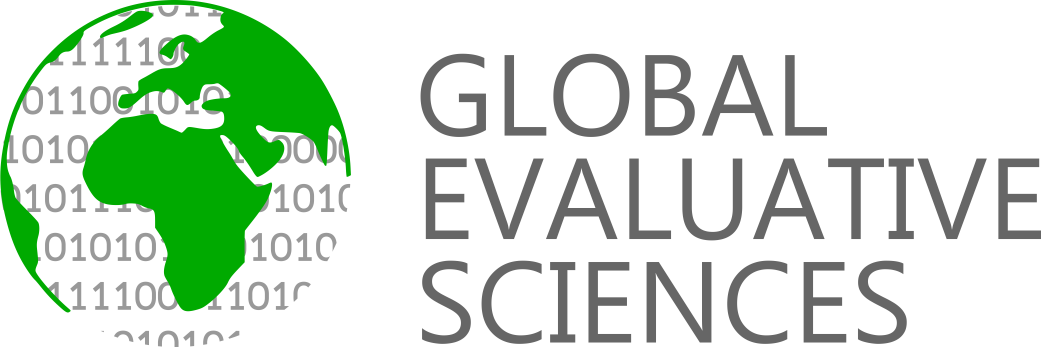

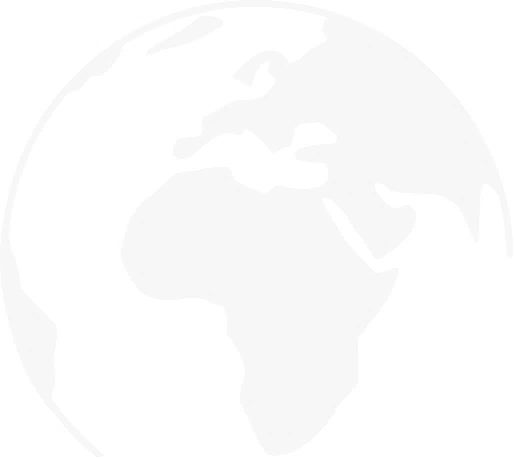


**Systematic Review to inform the World Health Organization Consolidated Antiretroviral Therapy Guidelines:**

**Systematic Literature Review Protocol –**

**Effect of frequency of clinic visits and medication pick-up on HIV care**

Version 2

Prepared by Global Evaluative Sciences

for

the World Health Organization

February 28, 2015

**Administrative structure**

Sponsor World Health Organization,

20 Avenue Appia,

CH-1211 Geneva 27,

Switzerland

Operator Edward Mills (project leader; [emills@geshealth.com](mailto:emills@geshealth.com))

Steve Kanters (statistical leader; [skanters@geshealth.com](mailto:skanters@geshealth.com))

M. Eugenia Socías (clinical leader, esocias@geshealth.com)

Global Evaluative Sciences

208-1505 W 2^nd^ Ave

Vancouver, British Columbia, Canada

V6H 3X4

Contents

**Tables** [**4**](#__RefHeading___Toc285724419)

Abbreviations [5](#__RefHeading___Toc285724420)

1. Introduction [6](#__RefHeading___Toc285724421)

2. Protocol [7](#__RefHeading___Toc285724422)

2.1. Research questions [7](#__RefHeading___Toc285724423)

2.2. Criteria for study inclusion [8](#__RefHeading___Toc285724424)

2.3. Literature search [9](#__RefHeading___Toc285724425)

2.3.1. Sources [9](#__RefHeading___Toc285724426)

2.3.2. Search strategy [9](#__RefHeading___Toc285724427)

2.4. Study selection [10](#__RefHeading___Toc285724428)

2.5. Study quality [11](#__RefHeading___Toc285724429)

2.6. Data extraction [11](#__RefHeading___Toc285724430)

2.6.1. Study characteristics [11](#__RefHeading___Toc285724431)

2.6.2. Intervention characteristics [12](#__RefHeading___Toc285724432)

2.6.3. Patient characteristics at baseline [12](#__RefHeading___Toc285724433)

2.6.4. Outcome measures [12](#__RefHeading___Toc285724434)

2.7. Analysis Plan [13](#__RefHeading___Toc285724435)

2.7.1. Fixed and random effects meta-analysis for AB trials [14](#__RefHeading___Toc285724436)

2.7.2. Meta-regression [14](#__RefHeading___Toc285724437)

2.7.3. Software [15](#__RefHeading___Toc285724438)

References [16](#__RefHeading___Toc285724439)

Appendix A [17](#__RefHeading___Toc285724440)

# Tables

Table 1: Scope of the literature review in PICOS form [8](#__RefHeading___Toc285724441)

Table 2: Systematic literature search [9](#__RefHeading___Toc285724442)

# Abbreviations

| ART | Antiretroviral therapy |
| --- | --- |
| ARV | Antiretrovirals |
| CFB | Change from baseline |
| CI | Confidence interval |
| CROI | Conference on Retroviruses and Opportunistic Infections |
| CTX | Co-trimoxazole |
| GRADE | Grading of Recommendations Assessment, Development and Evaluation |
| IAS | Conference on HIV Pathogenesis, Treatment and Prevention |
| IDU | Injection drug users |
| INH | Isonicotinylydrazine |
| IPT | Isoniazid preventative therapy |
| LTFU | Lost to follow-up |
| MCMC | Markov chain Monte Carlo |
| MSM | Men who have sex with men |
| PICOS | Population, interventions, comparisons, outcomes, study design |
| PLHIV | People living with HIV |
| RCT | Randomized controlled trials |
| RLS | Resource limited settings |
| SD | Standard deviation |
| SE | Standard error |
| SLR | Systematic literature review |

# Introduction

The recent scale-up of antiretroviral treatment (ART) programs in resource-limited-settings (RLS) has resulted in more than 12 million people living with HIV (PLHIV) receiving life-saving ART.[^1^](#_ENREF_1) As guidelines allow for people to get on treatment at earlier and earlier stages of their disease, there continues to be a need to put more and more people on treatment. Beyond financial limitations, important barriers to the continued expansion of ART initiation are limited clinical capacity and a lack of skilled healthcare workers. Various methods have been proposed and used to overcome this barrier, such as task-shifting and use of generic drugs. However, steps taken to reduce clinical requirement must not come at the expense of patient health. Optimal health, clinical and social outcomes requires early diagnosis, timely linkage and initiation of ART, and consistent adherence to ART.^2,3^ The discontinuation of ART can lead to drug resistance, AIDS-related illnesses and death, and disruption. The discontinuation of ART can lead to drug resistance, AIDS-related illnesses and death, and disruptions in care (e.g., missed visits) that undermine both social (e.g., acceptance of positive status) and clinical outcomes.^4-9^

Most ART programmes in RLS follow the World Health Organization (WHO) ART guidelines, which are based on a public health approach.^13^ In the case of RLS, PLHIV often have to come to the clinic monthly for check-ups and to pickup medication. At the same time, PLHIV often face multiple competing needs and logistical challenges that impede their ability to adhere to clinic visits and/or treatment with ART. This may be particularly true for individuals who have to travel long distances and deal with high transport costs.^14,15^

Given the importance of retention along the cascade, interventions that encourage continuous engagement while addressing the very real logistical challenges PLHIV face are clearly needed. Accordingly, the WHO is interested in evaluating the whether the frequency of expected clinical visits and/ medication pickup impacts on programme and patient outcome. This document outlines a protocol for a systematic literature search to identify and select relevant studies to answer the specific questions described below.

# Protocol

## Research questions

The specific research questions of the project are:

1. Among PLHIV, do less frequent clinic visits lead to comparable programme and patient outcomes compared to monthly visits? [Reference number: **F1.1**]
2. Among PLHIV, does less frequent pickup of ARV, CTX or IPT lead to comparable programme and patient outcomes compared to monthly pick up? [Reference number: **F1.2**]

## Criteria for study inclusion

The research questions contain elements that need to be defined before the start of the collection of evidence: population, interventions, comparisons, outcomes, and study design (PICOS criteria). **Table 1** describes these criteria for the combination of the two research questions in this systematic literature review, which will guide the identification and selection of studies that are relevant for the evidence synthesis.

Table 1: Scope of the literature review in PICOS form

| **Criteria** | **Definition** |
| --- | --- |
| **Population** | People living with HIV |
| **Interventions** | - Less frequent clinic visits (intervals that are greater than one month between visits) - Less frequent antiretroviral, co-trimoxazole and/or isoniazid preventative therapy pick-ups (intervals that are greater than one month between pickups) |
| **Comparator** | - Monthly clinic visits (or multiple visits per month) - Monthly antiretroviral, co-trimoxazole and/or isoniazid preventative therapy pick-ups (or multiple pick-ups a month) |
| **Outcomes** | - Mortality - Morbidity - Treatment adherence - Retention (pre- and post-ART initiation) - Patient and provider acceptability - Cost (including opportunity costs) - Transfer out of programmes - TB incidence* - Incidence of INH-related adverse events* |
| **Study design** | Randomized controlled trials and observational studies |

* These outcomes only apply to question F1.2.

Evidence from RCTs is expected to be limited for these research questions. Therefore, observational study designs will be included in the SLR. These will include cohort studies, case control studies, programme studies and cross-sectionals. Case reports and case series will be excluded.

This systematic literature review will include reviews and analyses for a variety of sub-populations: PLHIV adherent and stable on ART, PLHIV presenting late in the course of HIV infection, PLHIV failing first line ART, PLHIV presenting early, adults, adolescents, children, pregnant and breastfeeding women, rural versus urban population, PLHIV not on ART (i.e., CTX and IPT), and high income versus LMICs. These sub-populations and sub-analyses will not be searched on outside of the principal SLR. Our search strategy (specified below) will have the appropriate breadth to capture all the specified sub-populations.

## Literature search

### Sources

A comprehensive systematic search of the literature will be conducted using the following databases: EMBASE, MEDLINE and Cochrane Central Register of Controlled Trials. Conference abstracts provided through the EMBASE search, as well as the International AIDS conference (AIDS), the annual Conference on Retroviruses and Opportunistic Infections (CROI), and the [conference on HIV Pathogenesis, Treatment and Prevention (IAS)](http://www.ias2015.org/) will also be reviewed to determine if there were relevant studies recently completed (conference abstracts will be restricted to within three years). Additionally, hand searches of the bibliographies of published systematic reviews and health technology assessments will be performed.

### Search strategy

The following search strategy, provided in **Table 3**, will be used for the combined search strategy for Medline and EMBASE using OVID. The same search strategy will be adapted to the other search engines listed above.

Table 2: Systematic literature search

| **c** | **Term** | **Comments** |
| --- | --- | --- |
|  | exp HIV/ OR exp HIV Infection/ | HIV/AIDS terms |
|  | (HIV Infections OR hiv?1* OR hiv?2* OR HIV infect* OR human immuno?deficiency virus OR human immune?deficiency virus).ti,ab. |
|  | ((human immun*) AND (deficiency virus)).ti,ab. |
|  | (acquired immuno?deficiency syndrome OR AIDS OR acquired immunedeficiency syndrome OR acquired immune deficiency).ti,ab. |
|  | ((acquired immun*) AND (deficiency syndrome)).ti,ab. |
|  | or/1-5 | **Population Final** |
|  | exp Appointments and Schedules/ | Frequency of visit and pickup |
|  | ((frequen* OR schedule* OR month* OR expect*) ADJ7 (visit* OR appoint* OR attend* OR follow-up*)).ti,ab,kw. |
|  | ((frequen* OR schedule* OR month* OR expect*) ADJ7 (pick?up* OR pick-up* OR deliver* OR pharma*)).ti,ab,kw. |
|  | exp Antiretroviral Therapy, Highly Active/ | ART and treatment |
|  | exp Anti-HIV Agents/ |
|  | exp Trimethoprim-Sulfamethoxazole Combination/ |
|  | (Septrin* or Bactrim* or Bactrimel* or Biseptol* or Cotrim* or Resprim* or Septra* or Sulfatrim* or Trisul* or Polytrim*).ti,ab. |
|  | co?trimoxazole.ti,ab. |
|  | (sxt or tmp-smx or tmp-smz or tmp-sulfa).ti,ab. |
|  | exp isoniazid/ |
|  | (Hydra* or Isovit* or Laniazid* or Nydrazid*).ti,ab. |
|  | (isonicotinylhydrazine or inh).ti,ab. |
|  | (or/7-8) OR (9 AND (or/10-18)) | **Intervention and comparators final** |
|  | (Randomized Controlled Trial or Controlled Clinical Trial).pt. | Randomized controlled trial terms |
|  | (Clinical Trial or Clinical Trial, Phase II or Clinical Trial, Phase III or Clinical Trial, Phase IV).pt. |
|  | Multicenter Study.pt. |
|  | Randomized Controlled Trial/ or Randomized Controlled Trials as Topic/ or "Randomized Controlled Trial (topic)"/ |
|  | Controlled Clinical Trial/ or Controlled Clinical Trials as Topic/ or "Controlled Clinical Trial (topic)"/ |
|  | Clinical Trial/ or Phase 2 Clinical Trial/ or Phase 3 Clinical Trial/ or Phase 4 Clinical Trial/ |
|  | Clinical Trials as Topic/ or Clinical Trials, Phase II as Topic/ or Clinical Trials, Phase III as Topic/ or Clinical Trials, Phase IV as Topic/ |
|  | "Clinical Trial (topic)"/ or "Phase 2 Clinical Trial (topic)"/ or "Phase 3 Clinical Trial (topic)"/ or "Phase 4 Clinical Trial (topic)"/ |
|  | (Nonrandom* or non random* or non-random* or quasi-random* or quasirandom*).ti,ab,hw. | Observational study design terms |
|  | cohort studies/ or cohort analysis/ |
|  | longitudinal studies/ or longitudinal study/ |
|  | prospective studies/ or prospective study/ |
|  | follow-up studies/ or follow up/ or followup studies/ |
|  | retrospective studies/ or retrospective study/ |
|  | observational study/ |
|  | quasi experimental methods/ or quasi experimental study/ |
|  | (quasi adj (experiment or experiments or experimental)).ti,ab. |
|  | ((non experiment or nonexperiment or non experimental or nonexperimental) adj3 (study or studies or design or analysis or analyses)).ti,ab. |
|  | or/20-37 | **Study design final** |
|  | 6 and 19 and 38 | **Complete search** |
|  | 39 not (review or letter or meta-analysis or case report* or case series or posters or News or Newspaper article or meeting abstracts or lectures or interview or historical article or handbooks or guidelines or guidebooks or essays or editorial or comment or clinical conference or catalogs).pt. | Remove unwanted publication types |

## Study selection

Two investigators working independently will scan all abstracts and proceedings identified in the literature search. The same two investigators will independently review abstracts and proceedings potentially relevant in full-text. If any discrepancies occur between the studies selected by the two investigators, a third investigator will provide arbitration.

## Study quality

We will employ the Grading of Recommendations Assessment, Development and Evaluation (GRADE) system for rating overall quality of evidence.^16-21^ Three of our team members (Mills, Thorlund, Schünemann) have been involved with developing GRADE since its inception. GRADE provides a framework for interpreting the strength of inference from a review. Dr. Schünemann, in particular, has worked extensively with WHO to implement the GRADE criteria within the guideline decision-process. Most recently, GRADE has issued guidance on network meta-analysis (see section 2.7.1).^22^ For each outcome, randomized trials begin as high quality evidence, but may be rated down by one or more of five categories of limitations: (1) risk of bias, (2) consistency, (3) directness, (4) imprecision, and (5) reporting bias. The quality of evidence for each main outcome can be determined after considering each of these elements, and categorized as either **high** (We are very confident that the true effect lies close to that of the estimate of the effect), **moderate** (We are moderately confident in the effect estimate: The true effect is likely to be close to the estimate of the effect, but there is a possibility that it is substantially different), **low** (Our confidence in the effect estimate is limited: The true effect may be substantially different from the estimate of the effect ), or **very low** (We have very little confidence in the effect estimate: The true effect is likely to be substantially different from the estimate of effect).^23^ A GRADE is presented in **Appendix A**. GRADE tables will be filled out and provided as part of the final report. In the case of disagreement between reviewers, these will be resolved by discussion and, if necessary, the study authors will be contacted to resolve any uncertainties.

## Data extraction

Two investigators working independently will extract data on study characteristics, interventions, patient characteristics at baseline, and outcomes for the study populations of interest for the final list of selected eligible studies. Any discrepancies observed between the data extracted by the two data extractors will be resolved by involving a third reviewer and coming to a consensus. Data will be stored and managed in Microsoft Excel Workbooks with sheets corresponding to the different information categories.

### Study characteristics

Regarding baseline study design components we will extract the following information:

- Study design (e.g. cohort study, case-control)
- Study inclusion criteria
- Study exclusion criteria
- Study setting and location (e.g., rural, urban)
- Intervention
- Comparator
- Follow-up period
- Sample size at baseline and follow-up by intervention group
- Study quality items (GRADE)

### Intervention characteristics

The following information will be extracted regarding interventions:

- Visit frequency
- Medication pickup frequency
- Eligibility criteria

### Patient characteristics at baseline

Regarding baseline patient characteristics we will extract data for the following variables:

- Age (mean/median, standard deviation [SD])
- Sex (n, %)
- Ethnicity
- % rural
- Median travel time to clinic
- Level of care of facility (e.g., primary health centre, district hospital, dispensary)
- CD4 cell count (cells/µL)
- HIV RNA level (log10 copies/mL)
- Duration of HIV infection (years)
- Time since ART initiation
- AIDS-Defining illnesses at baseline
- Risk factors (IDU, MSM, etc.)
- Proportion of pregnant women
- Proportion of TB patients
- Proportion of HBV patients
- Proportion of HCV patients

### Outcome measures

Outcome measures for the following outcome measures will be extracted for the reported populations or subgroups of interest:

- Mortality
- Morbidity (other illnesses)
- Retention or attrition
- ART adherence
- INH-related events
- Viral suppression
- Change in CD4 cell count (cells per µL)
- Patient and provider acceptability
- Patients who transferred out, stopped, ART other medication
- Costs including opportunity costs (e.g., money saved by not missing work to come for frequent visits, cost-effectiveness)

And additionally for research question F1.2:

- TB co-infection
- AIDS-Defining illnesses

For each of continuous outcome the mean change from baseline (CFB), and number and % patients at target as defined in study at the end of the randomized phase will be extracted, along with corresponding sample size, SD for mean CFB and measures of uncertainty (i.e. standard error (SE), 95% confidence intervals, and p-value) for all relevant intervention groups.

If the mean CFB is not provided, we will extract the score at follow-up time point of interest and the baseline score, and calculate the change. In such cases, the SE of the change from baseline will be calculated by assuming a conservative 0.50 correlation. However, if estimates of the correlation between baseline and end of treatment/follow-up are available, we will use this value to calculate the standard error.

If the se is not reported it will be calculated according to the following hierarchy: based on the reported 95% CI by intervention group; SD by intervention group along with sample size; 95% CI of the difference between intervention groups; p-values by intervention groups; p-values for the difference between intervention groups.

## Analysis Plan

Pairwise meta-analysis will be employed given that there are only two treatment options per research question. However, given that frequency may differ in length, meta-regression will be used to account for difference in frequencies if sufficient data exist. Analyses will be conducted by either using the traditional frequentist approach or by using Bayesian methods. Bayesian methods will particularly be used if hierarchical modeling is called for. In this section we describe the concepts and models that will be employed in our analyses.

### Fixed and random effects meta-analysis for AB trials

In equation 1 the fixed effects meta-analysis model for RCTs is presented.

(1)

reflects the ‘underlying’ outcome for treatment *k* in study *j* and the link function to transform this outcome to a normally distributed scale (such as log of the odds or change from baseline, or log hazard rate): where is the link function and are the unknown parameters of the likelihood function. represents this (transformed) outcome in trial *j* with comparator treatment A. *d* is the underlying *treatment effect* of B versus A on a normal scale that is the same for each study *j*. With the random effects meta-analysis model is the trial-specific relative treatment effect of B relative to A. These trial-specific relative effects are drawn from a random effects distribution

(2)

### Meta-regression

If there are a sufficient number of studies in the network, it may be possible to perform a meta-regression analysis where the relative treatment effect of each study is a function of not only a treatment comparison of that study but also an effect modifier. In other words, with a meta-regression model we estimate the pooled relative treatment effect for a certain comparison based on the available studies, adjusted for differences in the level of the effect modifier between studies. Meta-regression analysis can help explain between-study heterogeneity and minimize (bias) in indirect comparisons due to transitivity violations.

### Software

The parameters of the different models will be estimated within a Bayesian framework using a Markov Chain Monte Carlo (MCMC) method as implemented in the WinBUGS/OpenBUGS software package.[^24^](#_ENREF_24) A first series of iterations from the WinBUGS/OpenBUGS sampler will be discarded as ‘burn-in’ and the inferences will be based on additional iterations using two chains. Convergence of the chains will be confirmed by the Gelman-Rubin statistic. All analyses will be condicted in R version 3.1.2, which will be used to access WinBUGS/OpenBUGS.

# References

# Appendix A

GRADE scoring systems for assessment of study quality

| Type of evidence | | |
| --- | --- | --- |
| Initial score based on type of evidence | +4 | RCTs/ SR of RCTs, +/– other types of evidence |
|  | +2 | Observational evidence (e.g., cohort, case-control) |
| **Quality** | | |
| Based on | Blinding and allocation process | |
|  | Follow-up and withdrawals | |
|  | Sparse data | |
|  | Other methodological concerns (e.g., incomplete reporting, subjective outcomes) | |
| Score | 0 | No problems |
|  | –1 | Problem with 1 element |
|  | –2 | Problem with 2 elements |
|  | –3 | Problem with 3 or more elements |
| **Consistency** | | |
| Based on | Degree of consistency of effect between or within studies | |
| Score | +1 | Evidence of dose response across or within studies (or inconsistency across studies is explained by a dose response); also 1 point added if adjustment for confounders would have increased the effect size |
|  | 0 | All/most studies show similar results |
|  | –1 | Lack of agreement between studies (e.g., statistical heterogeneity between RCTs, conflicting results) |
| **Directness** | | |
| Based on | The generalisability of population and outcomes from each study to our population of interest | |
| Score | 0 | Population and outcomes broadly generalisable |
|  | –1 | Problem with 1 element |
|  | –2 | Problem with 2 or more elements |
| **Effect size** | | |
| Based on | The reported OR/RR/HR for comparison | |
| Score | 0 | Not all effect sizes >2 or <0.5 and significant; or if OR/RR/HR not significant |
|  | +1 | Effect size >2 or <0.5 for all studies/meta-analyses included in comparison and significant |
|  | +2 | Effect size >5 or <0.2 for all studies/meta-analyses included in comparison and significant |

**The final GRADE score:** we use 4 categories of evidence quality based on the overall GRADE scores for each comparison: high (at least 4 points overall), moderate (3 points), low (2 points), and very low (one or less).
